# Integrated lipidomic and transcriptomic profiling of imiquimod-induced psoriasiform dermatitis

**DOI:** 10.3389/fcell.2026.1881695

**Published:** 2026-09-25

**Authors:** Eva Wisniewski, Wenwen Du, Jenna A. Himelstein, Gergő Szanda, Taylor J. Woodward, Ken Mackie, Heather B. Bradshaw

**Affiliations:** 1 Gill Institute for Neuroscience, Indiana University, Bloomington, IN, United States; 2 Department of Psychological and Brain Sciences, Indiana University, Bloomington, IN, United States; 3 Center for Cannabis, Cannabinoids, and Addiction, Indiana University, Bloomington, IN, United States

**Keywords:** endocannabinoid system, psoriasiform dermatitis, skin compartmentalization, targeted lipidomics, transcriptomics

## Abstract

Psoriasis is a chronic inflammatory skin disease characterized by keratinocyte hyperproliferation and immune dysregulation. Emerging clinical and experimental evidence suggests that endogenous lipid (endolipid) signaling systems, including the endocannabinoid system (ECS), represent a promising therapeutic target to treat psoriasis; however, comprehensive characterization of small-molecule endolipids and related proteins in psoriatic skin and their relationship to systemic changes remains limited. Here, we used the imiquimod (IMQ)-induced mouse model of psoriasis to perform combined lipidomic and transcriptional profiling of endolipid signaling in both skin and plasma. Targeted lipidomics revealed a striking divergence between tissues: most endolipids increased in inflamed skin but decreased in plasma, including the canonical ECS lipids anandamide and 2-arachidonoylglycerol. In contrast, selected lipid species, including taurine-conjugated metabolites (both *N*-acyl taurines and bile acids), were elevated in both tissues, indicating pathway-specific regulation. Targeted transcriptional analysis of whole skin showed reduced expression of key endolipid biosynthetic enzymes (*Napepld, Dagla, Daglb*) and the cannabinoid receptor *Cnr1*, while *Cnr2* and ECS-related metabolic enzymes remained unchanged. Additional alterations were observed in transcripts involved in related endolipid signaling (*Trpv1, Trpv4, Ppara, Pparg, Gpr55*), bile acid metabolism (*Fxr, Bsep, Fabp4, Fabp5, Cyp27a1, Cyp8b1*), and inflammatory pathways (*Cox-2*). To resolve this apparent discrepancy between lipid levels and gene expression, we performed compartment-specific analyses of epidermal and dermal layers. These revealed a predominantly suppressive epidermal response across multiple ECS-related proteins, contrasted by a more variable dermal profile with selective preservation or upregulation, particularly of *Cnr2*. Together, these findings demonstrate that psoriasiform inflammation is associated with compartment-specific remodeling of endolipid signaling across skin and systemic compartments, underscoring the functional heterogeneity of epidermal and dermal layers. This dataset provides novel insights into the dysregulation of endolipid signaling systems in psoriasis and provides a foundation for the development of spatially informed, lipid-based therapeutic strategies.

## 1 Introduction

Psoriasis is a chronic, immune-mediated skin disease affecting approximately 2%–3% of the global population (Crow, 2012). Emerging clinical evidence suggests that cannabinoids may have therapeutic potential in this disease: topical cannabidiol (CBD) has been reported to improve psoriatic lesions and reduce symptoms in human studies, suggesting a therapeutic role for the endocannabinoid system (ECS) (Friedman et al., 2020; Palmieri et al., 2019; Sheriff et al., 2020; Stanescu et al., 2024). These observations highlight endogenous lipid (endolipid) signaling pathways as promising targets in psoriasis.

Clinically, psoriasis presents as erythematous, scaly plaques driven by keratinocyte hyperproliferation and immune cell infiltration (Nestle et al., 2009; Boehncke and Schön, 2015). At the molecular level, this process is sustained by inflammatory cytokines including IL-17, IL-22, IL-23, and TNF-α, which mediate pathogenic immune-keratinocyte crosstalk (Kamata and Tada, 2023; Potestio et al., 2024). The disease arises from a complex interplay of genetic and environmental factors, such as infection, stress, or mechanical injury (Nestle et al., 2009; Boehncke and Schön, 2015). Beyond cutaneous manifestations, psoriasis is associated with systemic comorbidities such as psoriatic arthritis, cardiovascular disease, metabolic syndrome, and depression, resulting in substantial physical, psychosocial, and economic burden (Boehncke et al., 2010; Dowlatshahi et al., 2014; Liu et al., 2022; Singh et al., 2017; Al Sawah et al., 2017; Fowler et al., 2008; Veale and Fearon, 2018). Although advances in targeted therapies have improved disease management, long-term treatment remains challenging, underscoring the need for safer and more accessible strategies, especially topical treatments. A deeper understanding of disease mechanisms is therefore essential to identify new therapeutic targets. In this context, alterations in endolipid metabolism have emerged as a hallmark of psoriatic skin. Previous lipidomic studies have largely focused on circulating lipids or immune cells, with limited characterization of endolipids in lesional skin (Zeng et al., 2017; Coras et al., 2019; Ambrożewicz et al., 2018; Wójcik et al., 2019; Rousel et al., 2024). Consequently, integrated analysis of these endolipid mediators across both skin and systemic compartments remains lacking (Boboryko et al., 2026).

The cutaneous ECS is part of an endolipid signaling network expressed in multiple skin cell types (Maccarrone et al., 2003; Czifra et al., 2012; Pandey et al., 2009; Ständer et al., 2005) that contributes to skin homeostasis. The canonical ECS comprises the cannabinoid receptors CB1 and CB2, endogenous ECS ligands, including anandamide (AEA) and 2-arachidonoylglycerol (2-AG), and the enzymes responsible for their synthesis and degradation (Kupczyk et al., 2009; Eagleston et al., 2018). In addition to these canonical ECS ligands, a broader class of structurally related bioactive endogenous lipids, fatty acids and their oxygenated metabolites, glucocorticoids, and bile acids are in our lipidomics screening library and are collectively referred to here as endolipids. Several of these endolipids share metabolic pathways and functional overlap with ECS components in ways that are known; however, there are likely additional pathways and overlaps that are unknown that contribute to endolipid signaling networks in the skin. Collectively, these ECS-related signaling systems regulate keratinocyte proliferation and differentiation, inflammatory responses, lipid metabolism, and sensory processes such as pain and itch (Oláh et al., 2016; Tóth et al., 2019; Cintosun et al., 2020; Ramer and Hinz, 2022).

Dysregulation of ECS components has been reported in psoriasis, including altered expression of ECS-related genes (Nattkemper et al., 2018), altered receptor expression in lesional skin (Turk and Kirchhof, 2026), and changes in circulating endolipid levels (Ambrożewicz et al., 2018; Sorokin et al., 2018a). In preclinical models, endolipids, as well as synthetic and phytocannabinoids, exert anti-inflammatory, anti-proliferative, and anti-pruritic (anti-itch) effects in the skin (Eberlein et al., 2008; Schlosburg et al., 2009; Avila et al., 2020; Odan et al., 2012; Wilkinson and Williamson, 2007; Ramot et al., 2013; Simard et al., 2023; Kim et al., 2024), and experimental studies indicate a protective role for CB2 signaling in psoriasiform inflammation (Xu et al., 2025; Li et al., 2022; He et al., 2023).

Endolipid signaling is integrated into broader lipid mediator networks that likely play regulatory roles in disease progression. Bile acids have emerged as signaling molecules that can regulate inflammation and metabolism beyond their classical digestive roles. Conjugated bile acids can modulate N-acyl phosphatidylethanolamine-specific phospholipase D (NAPE-PLD), linking bile acid signaling to endolipid biosynthesis (Margheritis et al., 2016; Magotti et al., 2015). In addition, bile acids regulate nuclear receptors such as FXR and PPARα and influence pathways involved in inflammation and itch (Pineda Torra et al., 2003). We recently showed that endolipids are significantly dysregulated in adipose tissue in an FXR KO model that is exacerbated by obesity (Zhou et al., 2025). Modulation of bile acid signaling has also been reported to ameliorate psoriasiform dermatitis in experimental models (Shi et al., 2022; Yu et al., 2019). These observations suggest that inflammatory skin disease may involve coordinated remodeling of interconnected endolipid signaling pathways rather than isolated changes within a single system.

Topical application of imiquimod (IMQ) is a widely used murine model of psoriasis (Schön et al., 2021; Gangwar et al., 2022; Lin et al., 2025). IMQ acts as a TLR7/8 agonist and induces erythema, scaling, keratinocyte hyperproliferation, and immune cell infiltration, accompanied by activation of the IL-23/IL-17 axis (van der Fits et al., 2009; Swindell et al., 2011). Using this model, we evaluated the effects of psoriasiform inflammation on endolipid signaling systems in skin, including selective analyses of the epidermis and dermis, as well as in plasma, to better understand local and systemic endolipid mediator dynamics and their potential relevance to psoriasis.

## 2 Materials and methods

### 2.1 Subjects

Ten-week-old male C57BL/6J mice were purchased from The Jackson Laboratory (*Bar Harbor, ME, USA*) and acclimatized for one week prior to experimentation. Mice were housed under a 14/10-h light/dark cycle at room temperature (22 °C ± 1 °C) with *ad libitum* access to food and water. All experimental procedures were approved by the Institutional Animal Care and Use Committee (IACUC) of Indiana University (protocol number 24-037).

### 2.2 Imiquimod psoriasis model procedure

Psoriasis-like skin inflammation was induced using topical imiquimod (IMQ) as previously described (van der Fits et al., 2009). In brief, depilation of a 2 × 3 cm area on the dorsal back skin was achieved using Nair, then a daily dose of 62.5 mg of 5% IMQ cream (*Taro Pharmaceuticals, Israel*) was applied to the back and right ear for six consecutive days. Control mice underwent identical depilation but received no topical treatment. Given that the manufacturer’s vehicle was not commercially available and therefore another vehicle would not be a true control, we chose to compare endogenous lipid profiles between Nair + IMQ-induced psoriasiform and Nair alone skin rather than evaluating the contribution of another topical cream with different constituents than the manufacturer’s vehicle. Mice were evaluated daily for clinical signs of inflammation under isoflurane anesthesia. Back skin was scored for erythema (redness), scaling, and thickness using a semiquantitative scoring system based on the Psoriasis Area and Severity Index (PASI): 0 = none, 1 = slight, 2 = moderate, 3 = marked, and 4 = severe. In addition, body weight and the thickness of back and ear skin were measured daily using a digital micrometer (*Mitutoyo, Japan*).

On day 7 (after six treatments), mice were euthanized by isoflurane anesthesia and cervical dislocation. Two 4 mm-diameter punch biopsies were collected from the back skin and snap-frozen for lipidomic analysis and RNA extraction. Additional back and ear skin samples were fixed in 4% paraformaldehyde (PFA) for 24 h, then incubated in 30% sucrose for cryoprotection before cryosectioning. Heparinized blood was collected and centrifuged at 3,000 rpm for 10 min to isolate plasma, which was stored at −80 °C until further analysis.

For the follow-up experiment assessing mRNA expression in separated epidermal and dermal compartments, the same IMQ treatment protocol was applied to an independent cohort of 10 C57BL/6J mice. On day 7, dorsal skin from treated and control animals was harvested and processed exclusively for epidermis-dermis separation and subsequent mRNA analysis, without collecting additional tissues or plasma, to maximize RNA yield and preserve compartmental integrity.

### 2.3 Histological analysis

Cryoprotected tissues were embedded in M-1 embedding medium (*Thermo Fisher Scientific, Waltham, MA, USA*) and cryosectioned at 14 *µ*m thickness using a Leica CM1860 cryostat. Sections were stained using a hematoxylin and eosin (H&E) staining kit (*Abcam Limited, Cambridge, United Kingdom*), mounted with Permount™ mounting medium (*Fisher Chemical™, Waltham, MA, USA*), and imaged using a Pannoramic MIDI-III digital slide scanner (*3DHISTECH, Hungary, EU*).

### 2.4 Epidermis-dermis separation

After harvesting the back skin, the tissue was cut into smaller pieces and incubated overnight at 4 °C in 5 mg/mL Dispase II (*Sigma-Aldrich/Merck, Burlington, MA*) diluted in EpiLife medium (*Thermo Fisher Scientific, Waltham, MA*) with gentle rotation to facilitate enzymatic separation of epidermal and dermal layers. Following incubation, the epidermis was mechanically separated from the dermis using curved forceps under a stereomicroscope. Epidermal and dermal fractions were immediately transferred into TRI Reagent (*Zymo Research, Irvine, CA, USA*) for RNA stabilization, and RNA was extracted as described below.

### 2.5 Real-time quantitative PCR

Total RNA was extracted from back skin biopsies and epidermal/dermal layers using the Direct-zol RNA Miniprep Kit (*Zymo Research, Irvine, CA, USA*) according to the manufacturer's instructions. First-strand cDNA was synthesized from 3 *μ*g of total RNA using the RevertAid H Minus First Strand cDNA Synthesis Kit (*Thermo Fisher Scientific, Waltham, MA, USA*). Quantitative real-time PCR (qRT-PCR) was performed using TaqMan™ single-tube Gene Expression Assay in 96-well plates (*Applied Biosystems, Waltham, MA, USA*) on a QuantStudio™ 7 Flex Real-Time PCR System *(Thermo Fisher Scientific, Waltham, MA, USA*). For the duplex reactions, targets were dye-labeled with FAM™, and GAPDH-VIC™ was used as the internal reference gene, with two technical duplicates per sample.

TaqMan™ assay IDs targeting the mouse genes of interest were as follows:

**Table:** 

Gene	Assay ID
Abcb11	Mm00445168_m1
Abhd6	Mm00481199_m1
Abhd12	Mm00470489_m1
Cnr1	Mm01212171_s1
Cnr2	Mm00438286_m1
Cyp8b1	Mm00501637_s1
Cyp27a1	Mm00470430_m1
Dagla	Mm00813830_m1
Daglb	Mm00523381_m1
Dcn	Mm00514535_m1
Faah	Mm00515684_m1
Fabp4	Mm00445878_m1
Fabp5	Mm00783731_s1
Fabp7	Mm00445225_m1
Gapdh	Mm99999915_g1
Gpr55	Mm02621622_s1
Il1b	Mm00434228_m1
Il6	Mm00446190_m1
Krt10	Mm03009921_m1
Mgll	Mm00449274_m1
Napepld	Mm00724596_m1
Nr1h4	Mm00436425_m1
Ppap2a	Mm00477016_m1
Ppara	Mm00440939_m1
Pparg	Mm00440940_m1
Ptgs2	Mm00478374_m1
Trpv1	Mm01246300_m1
Trpv4	Mm00499025_m1

Relative gene expression was calculated using the 2^−ΔΔCt^ method (Livak and Schmittgen, 2001). Briefly, Ct values for each target gene, measured in technical duplicates, were normalized to the housekeeping gene GAPDH (ΔCt = Ct_target − Ct_GAPDH; measured simultaneously in the same well), and ΔΔCt was calculated relative to the control/calibrator sample or group (ΔΔCt = ΔCt_sample − ΔCt_control average), with expression reported as fold change (2^−ΔΔCt^). Outliers were identified using the ROUT method (Motulsky and Brown, 2006). Statistical analyses and graph generation were performed using GraphPad Prism (*GraphPad Software, San Diego, CA, USA*).

### 2.6 Lipid extraction and partial purification of plasma and skin

Lipid extraction and partial purification were performed as previously described (Zhou et al., 2025; Balaji et al., 2024). In brief, 50 *μ*L of plasma was added to 2 mL MeOH and 10 *μ*L deuterium-labeled anandamide (d8- AEA) and incubated on ice in the dark for 30 min. Skin punches were cut into four pieces on an ice-cold dissection plate, then added to 2 mL MeOH and 10 *μ*L d8-AEA and sonicated for 60 s, then incubated in the dark for 2 h. Organic supernatants for both tissues were isolated via centrifugation (19,000 g, 20 min, 20 °C), which were then added to 8 mL of HPLC-grade water to form a 20% organic solution. The solution was partially purified with 500 mg C-18 solid phase extraction columns (*Agilent, Santa Clara, CA, USA*) with final 1.5 mL elutions of 65, 75, and 100 percent methanol. The methanolic fractions were stored at −80 °C until mass spectrometry (MS) analysis.

### 2.7 Mass spectrometry and lipidomics analysis

Methanolic elutions were analyzed using HPLC/MS/MS as previously described (Zhou et al., 2025; Balaji et al., 2024) using the ABI 7500 (*Sciex, Framingham, MA, USA*) coupled to a Shimadzu LC system LC-40DX3 (*Kyoto, Japan*). Standard curves were generated by using purchased standards (*Cayman Chemical, Ann Arbor, MI, USA*) and those made in-house that were validated through NMR and MS analysis as previously described (Zhou et al., 2025; Balaji et al., 2024; Tan et al., 2006). One hundred and two endolipids were screened using Sciex OS analysis and peak-matching software (*Sciex, Framingham, MA, USA*) coupled to individual chromatographic validation auditing by research staff (*i.e.,* all chromatographic peaks were visualized and verified by an individual prior to data analysis). Statistical significance (p < 0.05) and trending (0.05 < p < 0.1) were differentiated in heatmaps that were generated to visualize changes in the concentration of each lipid analyte between the control and treatment groups as previously described (Leishman et al., 2016).

### 2.8 Statistical analysis

Statistical analyses were performed using GraphPad Prism (*GraphPad Software, San Diego, CA, USA) or Python (version 3.12.4*), as specified below. Unless otherwise indicated, data are presented as mean ± SEM, and *p* < 0.05 was considered statistically significant.

For longitudinal clinical measurements, including skin thickness and Psoriasis Area and Severity Index (PASI) scores, data from control and IMQ-treated mice (n = 10 per group) were analyzed using two-way repeated-measures ANOVA followed by Sidak’s multiple-comparisons test. Skin thickness measurements were normalized to the corresponding baseline value obtained on day 1 before the first treatment. Statistical significance is indicated as *p < 0.05, ***p < 0.001, and ****p < 0.0001. For RT-qPCR analyses, relative gene expression was calculated using the 2^−ΔΔCt^ method as described above. Comparisons between two independent groups were performed using the Mann–Whitney U test, with individual animals representing biological replicates (n = 10 mice per group for whole-skin analyses; n = 5 mice per group for compartment-specific analyses). Epidermal and dermal samples were analyzed separately, with IMQ-treated samples compared with their respective compartment-specific controls. Outliers were identified using the ROUT method as described above. Statistical significance is indicated as *p* < 0.05, **p < 0.01, ***p < 0.001, and ****p < 0.0001.

For lipidomic analyses, concentrations of individual analytes were determined from standard curves as described above and analyzed using Python (version 3.12.4). Control and IMQ-treated groups were compared using unpaired Student’s *t*-tests. Fold changes and Cohen’s *d* effect sizes were additionally calculated for each analyte. Analytes detected in fewer than four samples within a group were designated below analytical limits (BAL) and were not included in statistical comparison; analytes not detected in any samples were designated below detectable limits (BDL). Statistical significance was defined as *p* < 0.05, while 0.05 ≤ *p* < 0.10 was considered a statistical trend for visualization in the lipidomic heatmaps. Heatmaps display the direction and magnitude of fold changes together with statistical significance as described in the Mass Spectrometry and Lipidomics Analysis section.

## 3 Results

### 3.1 Imiquimod-induced psoriasis manifests as thickened, scaly, and erythematous skin in mice

Topical application of imiquimod (IMQ) reliably induced psoriasis-like dermatitis in C57BL/6J mice (Figure 1). As outlined in the experimental timeline (Figure 1A), mice received a daily dose of 62.5 mg 5% IMQ cream for six consecutive days, followed by tissue collection on day 7. Macroscopic examination revealed pronounced erythema, scaling, and plaque formation in IMQ-treated animals compared with controls (Figure 1C, top). Histological analysis of dorsal skin at day 7 further confirmed key features of psoriasiform dermatitis, including epidermal thickening (acanthosis), altered epidermal architecture, and dermal inflammatory cell infiltration (Figure 1C, bottom). These morphological changes were reflected in quantitative clinical parameters. Daily application of IMQ cream to the depilated back and right ear resulted in progressive increases in ear and back thickness, as well as elevated Psoriasis Area and Severity Index (PASI) scores for erythema, scaling, and thickening (Figure 1D). Clinical signs became evident by day 3 and continued to increase throughout the treatment period, with erythema reaching its peak on day 6. In contrast, untreated control animals showed no changes in skin thickness or PASI parameters.

**FIGURE 1:**
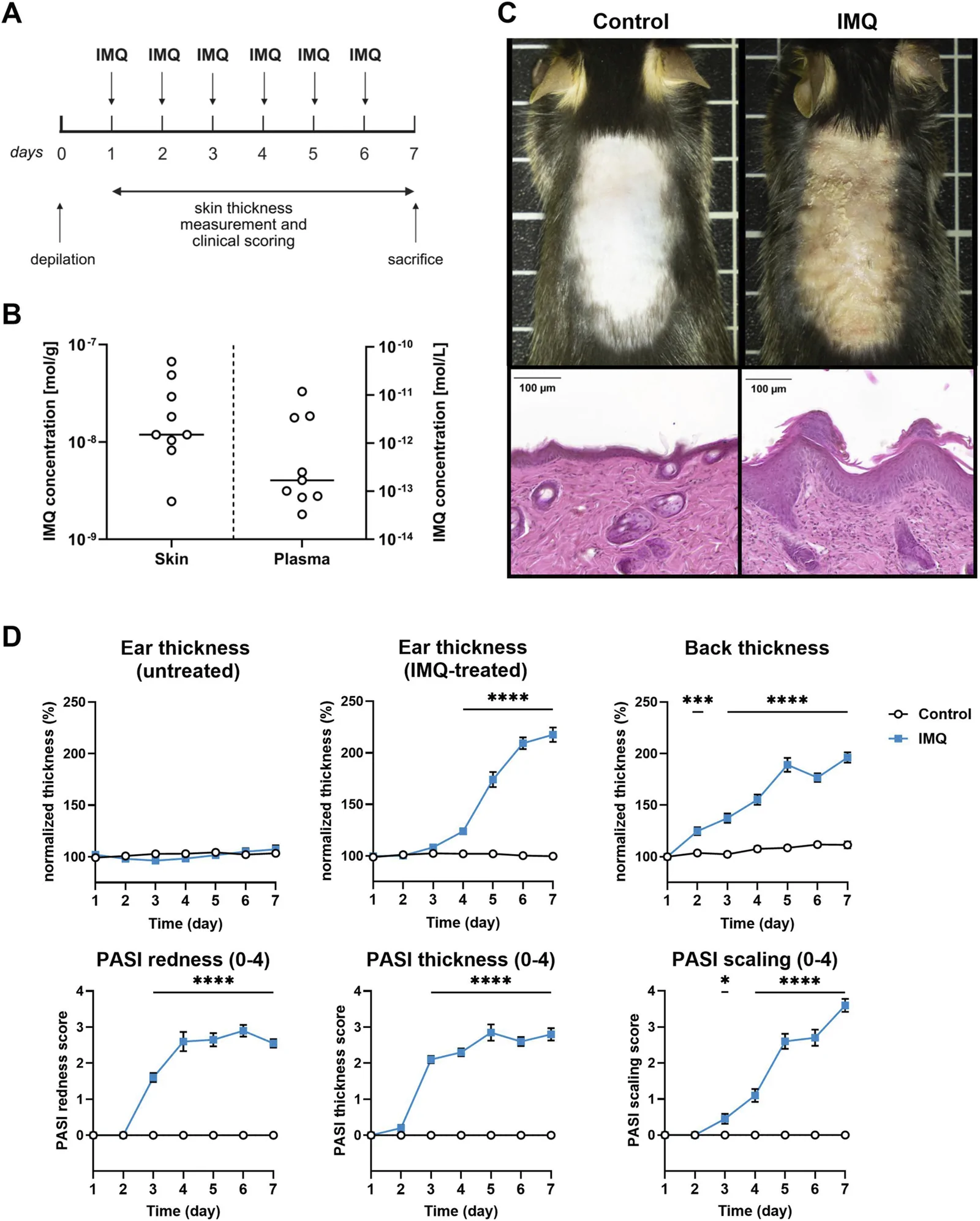
Clinical characterization of IMQ-induced psoriasiform dermatitis and tissue distribution of IMQ. **(A)** Experimental timeline. Mice received 62.5 mg daily topical 5% imiquimod (IMQ) application to the depilated back and right ear for 6 consecutive days. Skin thickness measurements and clinical scoring were performed daily, and tissues were collected on day 7. **(B)** Quantification of IMQ concentrations in skin and plasma 24 h after the final application (day 7) by mass spectrometry. IMQ accumulated in skin, whereas substantially lower levels were detected in plasma. Median is shown. **(C)** Representative dorsal skin images (top) and hematoxylin and eosin (H&E)-stained sections (bottom) from control and IMQ-treated mice at day 7. IMQ-treated skin exhibits erythema, scaling, epidermal thickening, and inflammatory cell infiltration. Scale bars: 100 µm. **(D)** Clinical disease progression during IMQ treatment. Normalized ear thickness (untreated and IMQ-treated ear), back skin thickness, and Psoriasis Area and Severity Index (PASI) parameters (erythema, thickness, and scaling; score 0–4) were assessed daily for 7 days. IMQ treatment resulted in progressive skin thickening and increased PASI scores compared with controls. Data are presented as mean ± SEM (n = 10 mice per group), thickness was normalized to that measured on day 1 prior to first treatment. Statistical analysis was performed using two-way repeated-measures ANOVA with Sidak’s multiple comparisons test. *p < 0.05, ***p < 0.001, ****p < 0.0001.

To confirm drug exposure, IMQ concentrations were quantified in skin and plasma 24 h after the final application on day 6. IMQ was readily detectable in both tissues but accumulated at markedly higher concentrations in skin than in plasma (Figure 1B), indicating predominantly local exposure. Notably, plasma IMQ levels did not correlate with those measured in skin across individual animals (Supplementary Figure S1). Together, these findings confirm robust induction of psoriasiform dermatitis in the cohort used for lipidomic and transcriptional analyses.

### 3.2 Lipidomics reveals complex tissue-specific changes of endolipids in a model of psoriasiform dermatitis

In the plasma, 83 of the 102 endolipids screened (81%) were detected at analytical limits, and of those, 29% remained unchanged. Of the 71% that significantly changed, 68% significantly decreased upon IMQ treatment. In the skin, 71 of the 102 endolipids screened (70%) were detected at analytical limits, and of those, 27% remained unchanged. Of the 73% that significantly changed, only 8% significantly decreased. Therefore, among IMQ-responsive endolipids, 92% increased in the skin, whereas only 32% increased in plasma. Figure 2 provides examples of individual endolipids in plasma and skin and control versus IMQ comparisons within tissue types (*e.g.,* fold change, *p*-values). The eCBs AEA and 2-AG are illustrated in 2A and B, respectively, and the AEA congener, *N*-oleoyl ethanolamine (OEA: Figure 2C), fits the profile of decreases in plasma and increases in skin. However, the OEA congener, *N*-oleoyl taurine (Figure 2D), does not fit the pattern in that the levels increase in both skin and plasma. Likewise, the bile acid cholic acid (CA; Figure 2E), decreases in both skin and plasma with IMQ treatment, but the taurine-derived CA metabolite, taurocholic acid (Figure 2F), increases in both tissues in the same way as *N*-oleoyl taurine.

**FIGURE 2:**
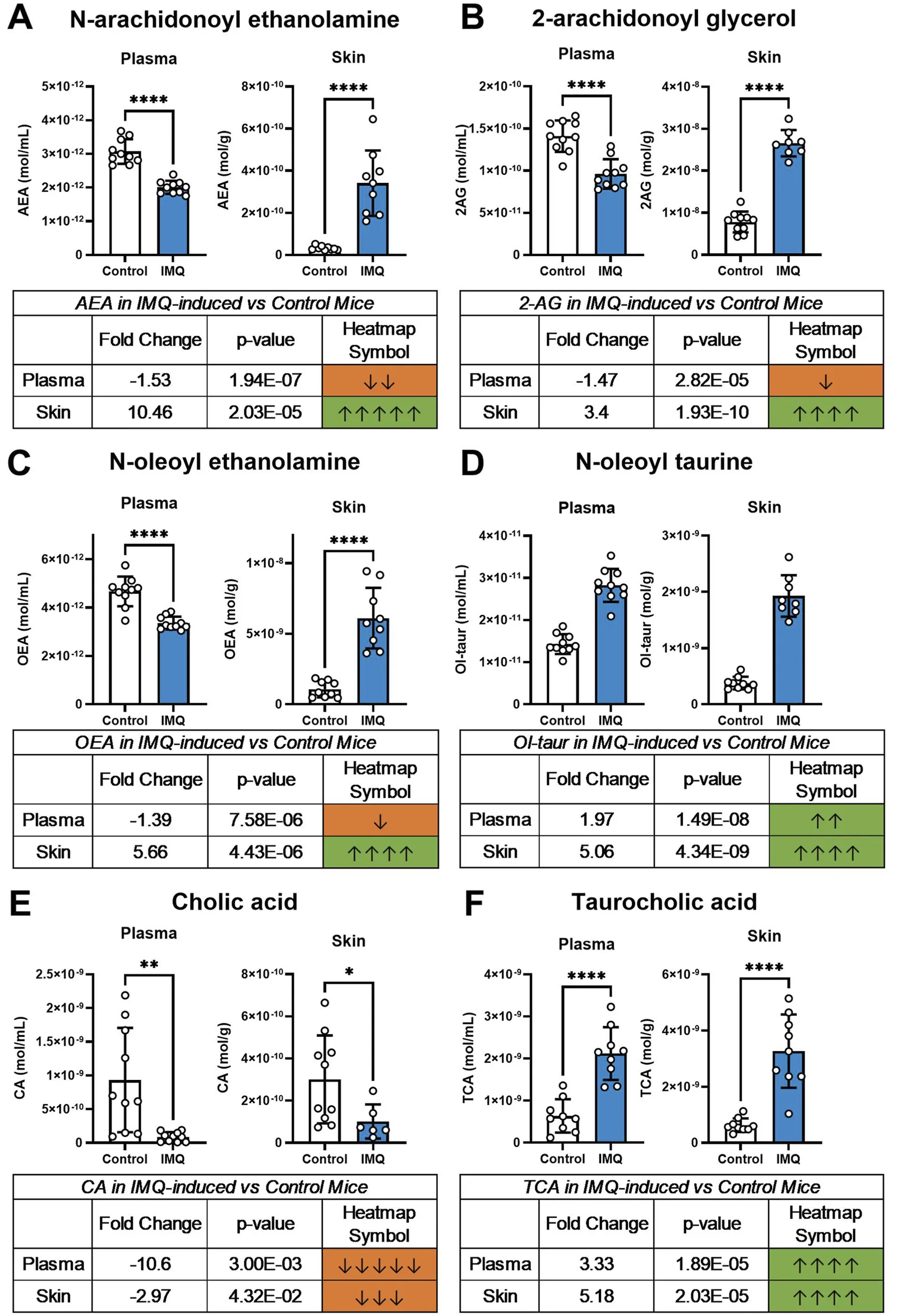
Representative bar graphs of endolipids in plasma and skin with heatmap icons. The bar graphs on the top of the examples are the mean SEM of the endogenous lipid levels in the treatment group (control in white and IMQ treatment in blue). **(A)** N-arachidonoyl ethanolamine (AEA; Anandamide); **(B)** 2-arachidonoyl glycerol (2-AG); **(C)** N-oleoyl ethanolamine (OEA); **(D)** N-oleoyl taurine (Ol-taur); **(E)** Cholic acid (CA); **(F)** Taurocholic acid (TCA). Tables underneath the bar graphs list the analytical data of the IMQ vs. control comparisons. See Methods and Figure 3 legend for description of Python data analysis for Fold change (denoted by arrows) and t-tests (colors denote significance levels and direction) to generate the heatmap colors and arrows.

Figure 3 is a heatmap summarizing all endolipids that showed significant changes in either skin or plasma following IMQ treatment, together with corticosterone and prostaglandin E_2_, whose levels remained unchanged. Endolipids are organized by lipid species type with cholesterol-based bile acids and corticosterone as one cluster, then by the fatty acid group in order of acyl chain length and level of desaturation (*e.g.*, palmitic, C16:0; stearic, C18:0; oleic, C18:1; linoleic, C18:2; arachidonic, C20:4; and docosahexaenoic, C22:6). See Methods and Figure 3 legend for a detailed description of the generation of the heatmaps. Also, see Supplementary Figure S2 for the whole dataset. These key patterns emerged in the data: 1) Overall, the longer chain, unsaturated fatty acids increased in the skin and decreased in the plasma, whereas the shorter, saturated acyl chain derivatives showed more variability. 2) Stearic acid derivatives were elevated in both skin and plasma with IMQ treatment except for the AEA congener, SEA. 3) The primary exception to the pattern other than acyl chain length was the addition of taurine or glycine as the amino acid conjugate. 4) The CA metabolite, TCA (formed through a conjugation of CA and taurine), is increased at the same rate as CA is decreased in both tissues. 5) Levels of corticosterone and prostaglandin E2 are unaffected by treatment in either tissue.

**FIGURE 3:**
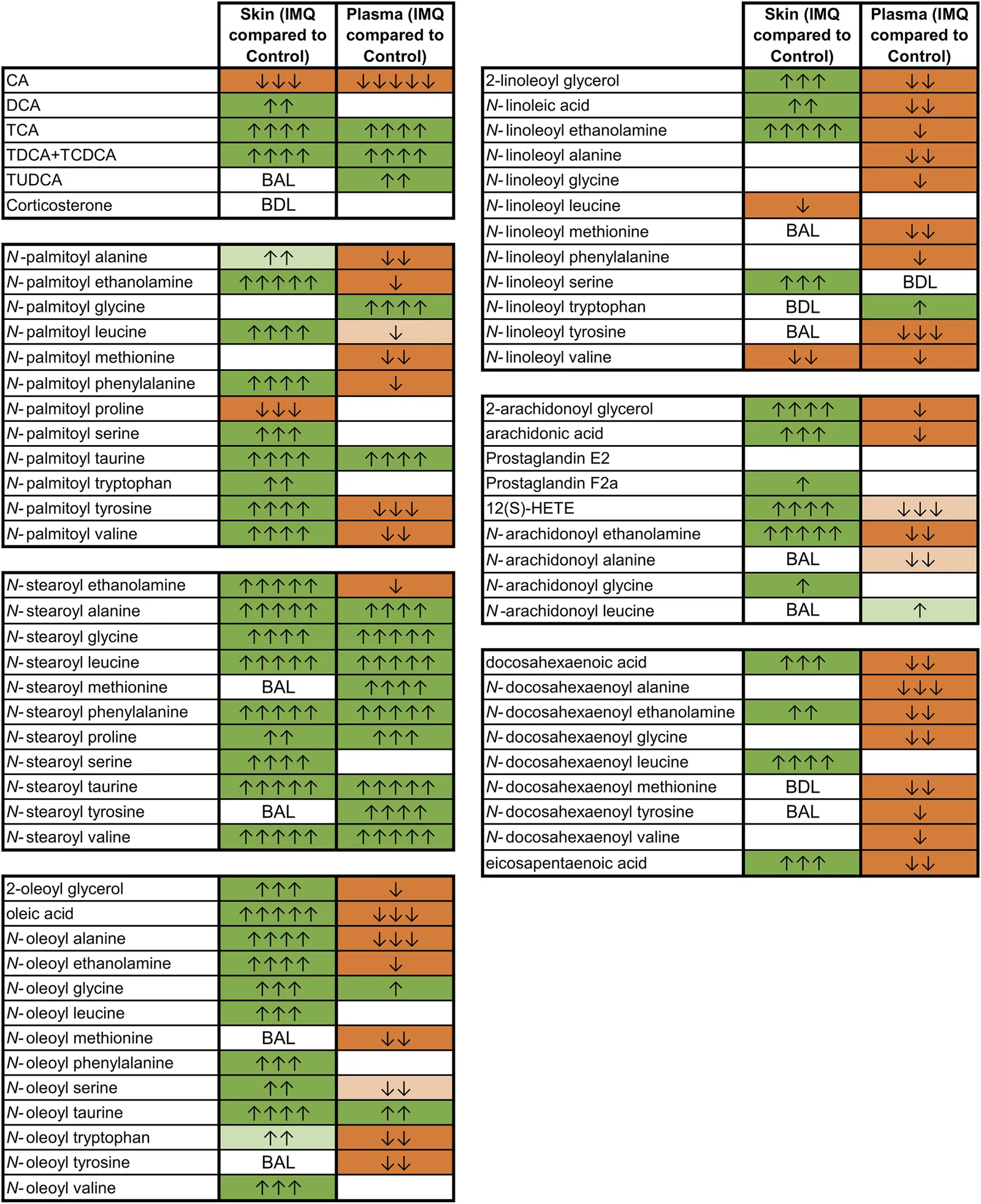
Heatmap of endolipid alterations in skin and plasma following IMQ treatment. Dark green represents significant increases after treatment (p ≤ 0.05), while light green represents significance levels of p ≤ 0.1–0.05. Dark orange represents significant decreases after treatment (p ≤ 0.05), while light orange represents significance levels of p ≤ 0.1–0.05. Fold change is indicated by the number of arrows, where 1 arrow corresponds to a 1–1.49-fold difference, 2 arrows to a 1.5–1.99-fold difference, 3 arrows to a 2–2.99-fold difference, 4 arrows to a 3–9.99-fold difference, and 5 arrows to a difference of tenfold or more. BAL (below analytical limits) refers to endolipids that were measured in at least one sample, but less than 4, making statistical analysis unreliable. BDL (below detectable limits) refers to endolipids that were not detected in any samples. Blank cells indicate that endolipids were present in at least 4 samples in each treatment group and that no significant differences were present. Abbreviations: CA, cholic acid; DCA, deoxycholic acid; TCA, taurocholic acid; TUDCA, tauroursodeoxycholic acid; TDCA, taurodeoxycholic acid; TCDCA, taurochenodeoxycholic acid; HETE, hydroxyeicosatetraenoic acid.

### 3.3 Psoriasiform inflammation induces coordinated transcriptional changes in endocannabinoid signaling and lipid-associated pathways

To determine whether the alterations in endolipid levels observed in the lipidomic analysis were accompanied by transcriptional changes of endolipid metabolic enzymes, mRNA expression of key ECS components and lipid-related genes was assessed in whole back skin by RT-qPCR. Levels of endocannabinoid-synthesizing enzymes were significantly reduced following IMQ treatment, including the *N*-acyl ethanolamine (NAE)-producing enzyme, *Napepld*, and the 2-acyl glycerol-generating diacylglycerol lipases *Dagla* and *Daglb* (Figure 4A). In contrast, expression of the primary degradative enzymes fatty-acid amide hydrolase (*Faah*) and monoacylglycerol lipase (*Mgll*) did not differ between IMQ-treated and control animals. Cannabinoid receptor *Cnr1* (CB1) expression was significantly decreased in psoriasiform skin, whereas *Cnr2* (CB2) expression remained unchanged (Figure 4A).

**FIGURE 4:**
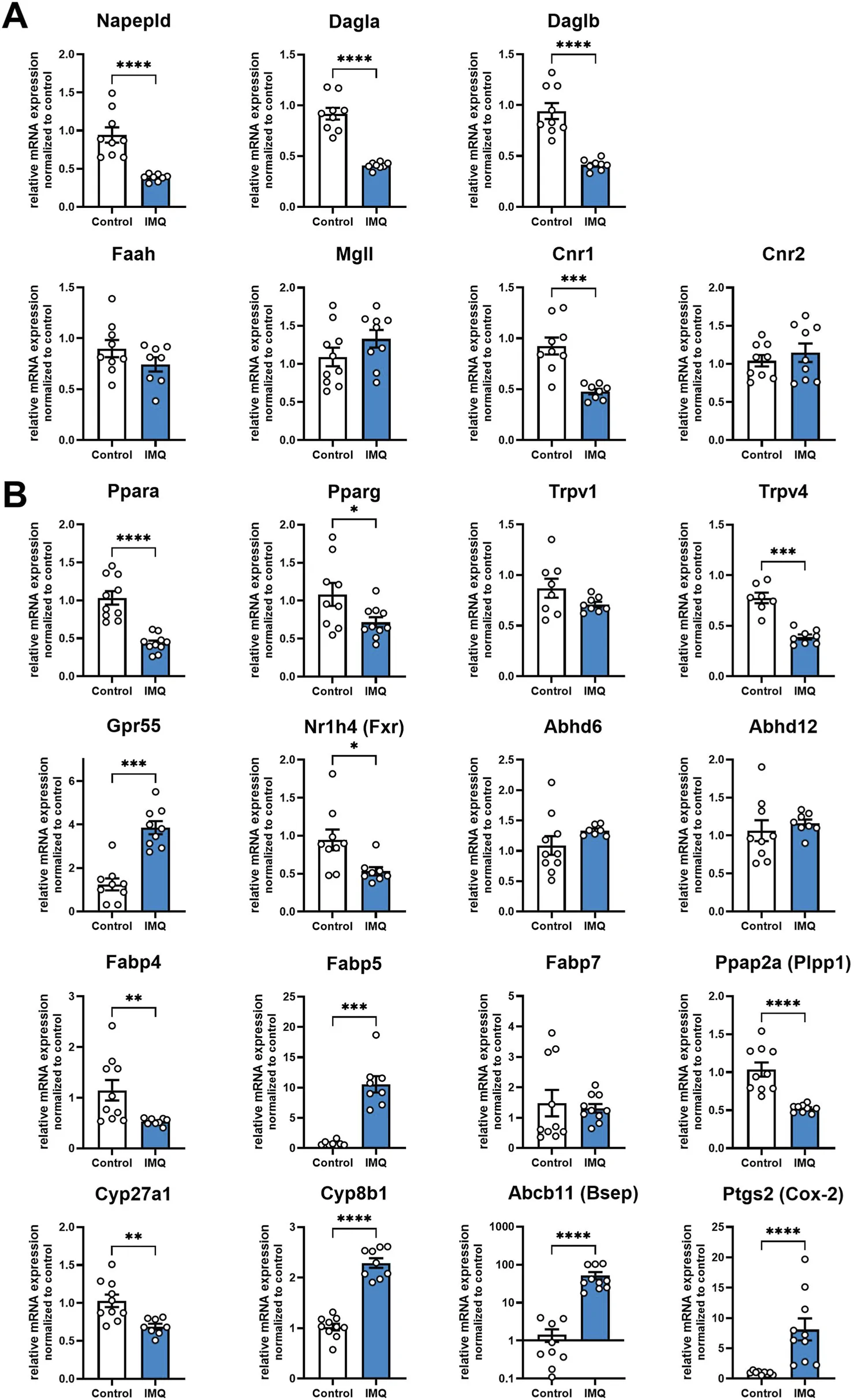
Psoriasiform inflammation drives coordinated transcriptional reprogramming of endocannabinoid and lipid pathways in the skin. Relative mRNA expression was quantified by RT-qPCR in back skin from control and IMQ-treated mice. **(A)** Canonical ECS components, including biosynthetic enzymes (*Napepld, Dagla, Daglb*), degradative enzymes (*Faah, Mgll*), and cannabinoid receptors (*Cnr1, Cnr2*); **(B)** Extended lipid-related pathways, including nuclear receptor signaling (*Ppara, Pparg, Nr1h4/FXR, Ppap2a*), non-classical cannabinoid-related receptors and channels (*Gpr55, Trpv1, Trpv4*), auxiliary endocannabinoid metabolism (*Abhd6, Abhd12*), intracellular lipid transport (*Fabp4, Fabp5, Fabp7*), bile acid synthesis and transport (*Cyp27a1, Cyp8b1, Abcb11*), and inflammatory lipid mediator production (*Ptgs2/COX-2*). Data are shown as individual animals with mean ± SEM (n = 10 per group). Statistical significance was assessed using the Mann–Whitney U test. Only statistically significant differences are indicated; absence of annotation denotes non-significant comparisons. *p < 0.05, **p < 0.01, ***p < 0.001, ****p < 0.0001.

To further explore molecular pathways contributing to the endolipid regulation observed in IMQ-treated mice, we reviewed a publicly available RNA-sequencing dataset of IMQ-induced dermatitis across multiple mouse strains (Swindell et al., 2017). Although the number of animals per group in that dataset was limited, the reported expression patterns for key ECS proteins were largely consistent with the changes observed in our cohort (Figure 4A). Based on this dataset and their biological relevance, we chose additional endolipid-related genes, involved in nuclear receptor signaling, non-canonical endocannabinoid pathways, lipid transport, bile acid metabolism, and inflammatory lipid mediator production (Figure 4B).

Within this extended panel, several nuclear receptor-associated pathways were significantly altered. Expression of *Ppara* and *Pparg* was reduced in IMQ-treated skin, as was the bile acid-sensing nuclear receptor *Nr1h4* (FXR), and *Ppap2a* was also significantly decreased. In contrast, expression of the non-classical cannabinoid-related receptor *Gpr55* was markedly increased. Among transient receptor potential (TRP) channels, *Trpv4* expression was significantly reduced, whereas *Trpv1* remained unchanged.

Genes involved in intracellular lipid handling showed differential regulation. *Fabp4* expression decreased, while *Fabp5* was strongly upregulated, and *Fabp7* did not change significantly. Expression of auxiliary endocannabinoid-metabolizing enzymes *Abhd6* and *Abhd12* was not significantly altered.

In pathways related to bile acid metabolism, expression of *Cyp8b1* and the bile salt export transporter *Abcb11* was robustly increased, whereas *Cyp27a1* expression was reduced. Finally, expression of *Ptgs2* (COX-2), a key enzyme in inflammatory lipid mediator synthesis, was markedly elevated in IMQ-treated skin.

Together, these findings show that IMQ-induced psoriasiform inflammation is accompanied by coordinated transcriptional changes extending beyond the canonical ECS, involving lipid transport, nuclear receptor signaling, bile acid metabolism, and inflammatory lipid mediator pathways. Because these analyses were performed in whole-skin samples, they do not resolve compartment-specific effects; however, given the distinct functional roles of epidermal and dermal compartments, differential regulation within these layers is likely and was therefore investigated in subsequent experiments (Figure 5).

**FIGURE 5:**
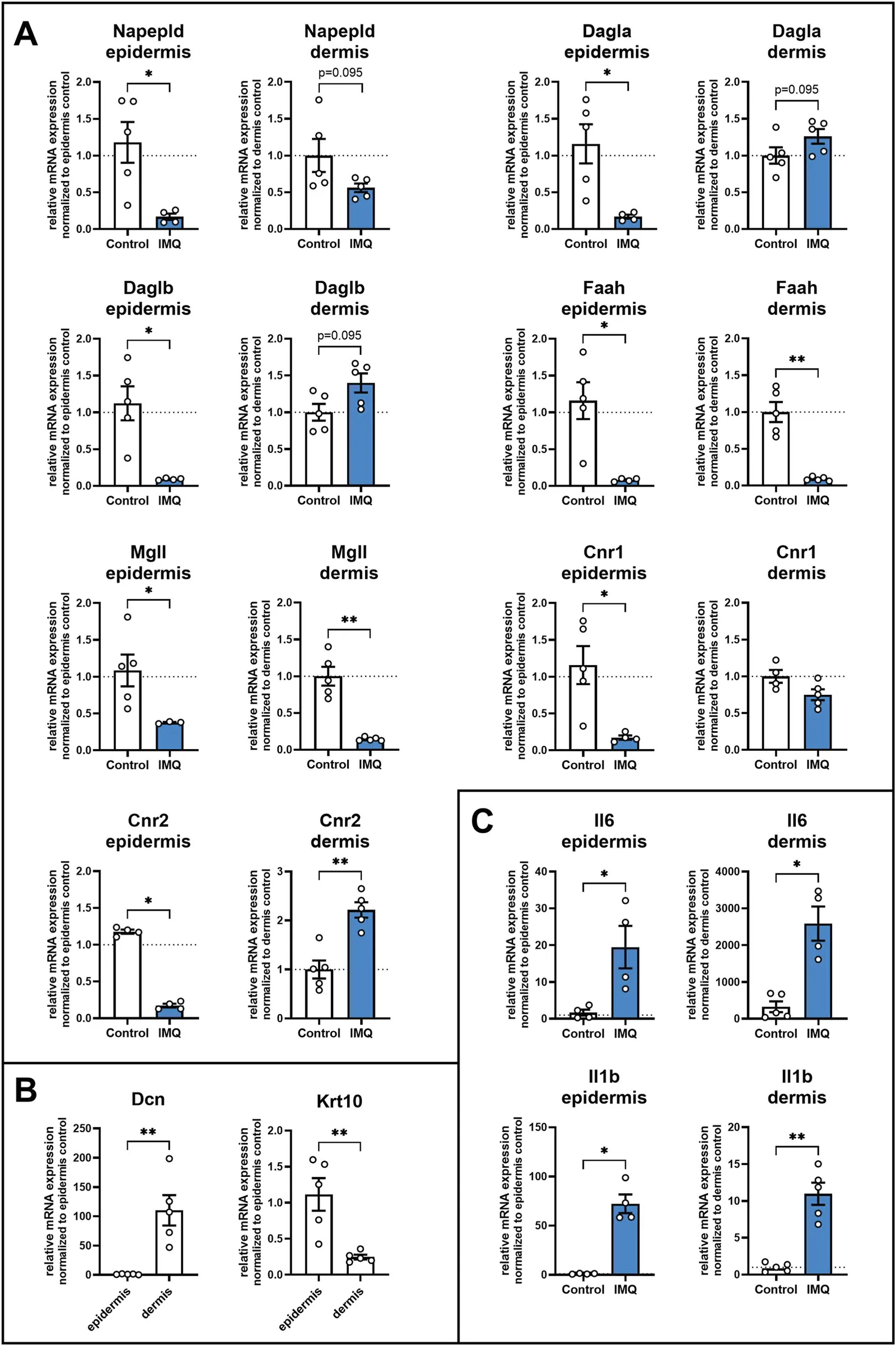
Layer-specific alterations in endocannabinoid system components in epidermis and dermis following IMQ treatment. Relative mRNA expression was quantified by RT-qPCR in enzymatically separated (Dispase II) epidermal and dermal fractions of control and IMQ-treated mice. Data within each skin layer are normalized to the corresponding control group. **(A)** Endocannabinoid system components, including biosynthetic enzymes (*Napepld, Dagla, Daglb*), degradative enzymes (*Faah, Mgll*), and cannabinoid receptors (*Cnr1, Cnr2*); **(B)** Validation of tissue separation using epidermal (*Krt10*) and dermal (*Dcn*) marker genes; **(C)** Inflammatory cytokines (*Il1b, Il6*). Data are shown as individual animals with mean ± SEM (n = 4–5 per group). Statistical analysis was performed separately within each skin layer to assess the effect of IMQ treatment (control vs. IMQ) using the Mann–Whitney U test. Comparisons between epidermal and dermal compartments were not performed. Only statistically significant differences are indicated (*p < 0.05, **p < 0.01).

### 3.4 Layer-specific regulation of endocannabinoid system components in epidermis and dermis

The whole-skin RT-qPCR analysis revealed an unexpected transcriptional pattern, with reduced expression of key endocannabinoid biosynthetic enzymes despite elevated endolipid levels in psoriatic skin (Figure 4). Given that skin is a heterogeneous tissue composed of distinct cellular compartments with different lipid profiles (Kendall et al., 2015), and that psoriasiform inflammation further alters this composition through keratinocyte hyperproliferation and immune cell infiltration, we next assessed whether epidermal and dermal layers exhibit differential transcriptional responses.

To address this, in an independent cohort, dorsal skin samples were subjected to enzymatic separation of epidermis and dermis before mRNA analysis. Successful separation was confirmed by enrichment of the dermal marker *Dcn* in the dermis and the epidermal marker *Krt10* in the epidermis (Figure 5B).

Analysis of endocannabinoid biosynthetic enzymes revealed a clear compartment-specific pattern. In the epidermis, expression of *Napepld*, *Dagla*, and *Daglb* was markedly reduced following IMQ treatment. In contrast, *Dagla* and *Daglb* expression was maintained or modestly increased in the dermis, whereas dermal *Napepld* showed a non-significant decrease (Figure 5A). A different pattern was observed for the degradative enzymes, with *Faah* and *Mgll* expression significantly reduced in both the epidermis and dermis following IMQ treatment (Figure 5A).

Cannabinoid receptor expression further highlighted this compartmentalization. *Cnr1* expression was substantially reduced in the epidermis, with comparatively minor changes in the dermis. In contrast, *Cnr2* expression decreased in the epidermis but was significantly increased in the dermal compartment following IMQ treatment (Figure 5A).

To confirm inflammatory activation within both compartments, expression of *Il1b* and *Il6* was assessed. Both cytokines were robustly induced in epidermal and dermal fractions (Figure 5C).

Together, these data demonstrate that IMQ-induced inflammation is associated with distinct, layer-specific regulation of ECS-related genes that is not captured in whole-skin analyses.

## 4 Discussion

Understanding how chronic skin inflammatory disorders like psoriasis are driven by alterations in endogenous lipid signaling remains an underexplored avenue for therapeutic development. By combining targeted lipidomics of unique, small-molecule endolipids with transcriptional profiling in the IMQ-induced psoriasis mouse model, we identified novel endolipid profiles in both skin and plasma and showed that endolipid-related enzymes and receptors are differentially regulated across skin compartments. These findings provide a framework for understanding how endolipid signaling is remodeled in psoriatic skin and highlight pathways with potential therapeutic relevance.

### 4.1 Human psoriasis disease markers and the intersection with the IMQ-mouse model

Human psoriasis is characterized by well-defined morphological and molecular features driven by immune cell-keratinocyte interactions. Histologically, lesions display epidermal hyperplasia (acanthosis) and immune cell infiltration. At the molecular level, disease pathogenesis is largely sustained by activation of the IL-23/Th17 axis, with cytokines such as IL-17A, IL-22, IL-23, and TNF-α promoting keratinocyte proliferation and inflammatory signaling. These processes are further supported by innate immune activation, including Toll-like receptor (TLR) pathways, and are associated with broad transcriptional and metabolic remodeling of the skin (Griffiths et al., 2021; Rendon and Schäkel, 2019).

The IMQ-induced mouse model recapitulates many of these hallmark features (Lin et al., 2025). Topical IMQ activates TLR7/8 signaling, leading to IL-23/IL-17-driven inflammation, epidermal hyperproliferation, and immune cell infiltration, closely mirroring key histological and immunological aspects of human plaque psoriasis (van der Fits et al., 2009). In addition, transcriptomic analyses have demonstrated substantial overlap between IMQ-induced gene expression signatures and those observed in human psoriatic lesions, particularly in pathways related to cytokine signaling, keratinocyte activation, and immune cell recruitment (Schön et al., 2021; Swindell et al., 2011; Swindell et al., 2017). These similarities support the translational relevance of the IMQ model for studying psoriasis-associated changes in endolipid signaling.

Within this translational framework, increasing attention has been directed toward lipid-mediated signaling pathways, particularly the cutaneous endocannabinoid system (ECS). ECS-related endolipids and their receptors regulate keratinocyte proliferation, immune responses, and sensory signaling, and growing evidence indicates that modulation of these pathways can attenuate inflammation and restore skin homeostasis (Ramer and Hinz, 2022; Martins et al., 2022; Río et al., 2018). Consistent with this, cannabinoid-based interventions have shown beneficial effects in both preclinical models and early clinical studies of psoriasis (Friedman et al., 2020; Palmieri et al., 2019; Stanescu et al., 2024; Eagleston et al., 2018; Ramer and Hinz, 2022; Wilkinson and Williamson, 2007; Li et al., 2022; Martins et al., 2022).

Lipidomic alterations are increasingly recognized as key features of psoriasis at both systemic and tissue levels. Circulating lipid profiles can reflect disease activity, serve as biomarkers or risk factors for comorbidities, and may be used to monitor therapeutic responses (Boboryko et al., 2026; Guo et al., 2024). At the same time, the lipid composition of the skin plays a critical role in barrier integrity and redox balance, while specific endolipid mediators actively contribute to inflammatory signaling and disease pathogenesis. A deeper understanding of the lipidomic “fingerprint” in psoriasis is therefore critical for improving prevention, diagnosis, and treatment strategies. However, previous human studies have largely focused on either systemic lipid changes (*e.g.*, plasma lipidomics of glycerophospholipids (Zeng et al., 2017), oxylipins/eicosanoids (Coras et al., 2019), long-chain fatty acids (Guo et al., 2024), ceramides (Rousel et al., 2024)) or cell- and compartment-specific analyses, such as isolated epidermis (Moon et al., 2013; Lew et al., 2006; Tyrrell et al., 2021), or immune cells (Ambrożewicz et al., 2018; Wójcik et al., 2019). Comprehensive analyses integrating endocannabinoid-related endolipids across both skin and plasma compartments have not been systematically conducted before.

By performing parallel lipidomic profiling in both skin and plasma, our study addresses this gap and provides an integrated view of local and systemic endolipid changes during psoriasiform inflammation. To complement these measurements and better interpret tissue-level changes, we assessed transcriptional regulation of ECS-related proteins in whole skin and in separated epidermal and dermal layers. This distinction is particularly relevant given the inherent heterogeneity of the skin. The skin comprises multiple metabolically active cell populations–including keratinocytes, fibroblasts, resident and infiltrating immune cells–that contribute differently to lipid signaling networks. Human studies have demonstrated that epidermis and dermis differ substantially in their bioactive lipid composition (Kendall et al., 2015), indicating that whole-skin measurements only represent a composite of functionally distinct compartments. Therefore, a layer-specific analysis provides critical context for interpreting bulk skin data and for understanding how endolipid signaling is differentially regulated across skin compartments during inflammation.

### 4.2 Lipidomics outcomes with the IMQ model elucidate novel endolipid signaling pathways

The pattern of endolipid regulation in the IMQ model illustrates that the directionality of change in plasma endolipids is the inverse of that for skin endolipids for ∼60% of those detected in the screening library. This finding raises the possibility that specific plasma endolipids that decrease significantly are being translocated to the skin because of cellular cues associated with chronic inflammation and that this translocation increases their levels in the skin. The molecular cues for this transfer may involve fatty acid binding proteins that were significantly modulated in the skin here (*e.g*., FABP4, FABP5). An alternative hypothesis is that the local production and metabolism of endolipids in the skin is modulated independently of systemic changes. A key difference in the hypothesis that decreases in plasma endolipids are driven by translocation to skin or tissue-specific enzymatic regulations is that stearic acid derivatives in the screen increased more than 3-fold in both tissues, with the single exception of *N*-stearoyl ethanolamine in plasma. The only other endolipids that had increases in both skin and plasma were specific members of the *N*-acyl glycine and *N*-acyl taurine groups. How these endolipid classes are signaling in this system is unknown, however, there are reports of *N-*acyl glycines acting on a range of GPCRs (McHugh et al., 2012; Drzazga et al., 2024; Console-Bram et al., 2017) and *N*-acyl taurines acting on ion channels including TRP channels (Saghatelian et al., 2006; Redmond et al., 2014).

Bile acids are a much smaller subset of endolipids in the screen, yet the profound effect on their levels in this model illustrates that multiple endolipid systems are engaged and likely intersect to drive the phenotype. IMQ treatment reduced cholic acid in both plasma and skin, while increasing taurine-conjugated bile acids, including taurocholic acid and tauro (cheno)deoxycholic acid. This shift is notable because bile acids are now recognized as signaling molecules with immunomodulatory functions (Cai et al., 2022; Fleishman and Kumar, 2024), and bile acid treatment has been reported to improve psoriasiform dermatitis through inhibition of IL-17A production and reduced CCL20-dependent trafficking (Shi et al., 2022). Relevant to our data, bile acids can allosterically regulate NAPE-PLD, and glyco- or tauro-dihydroxy conjugates can bind and activate this enzyme (Margheritis et al., 2016; Magotti et al., 2015). The expression profile of the stearic acid derivatives is like that of the bile acids, being the same in both plasma and skin (either increases or decreases in both), suggesting that the regulatory mechanisms of each of these specific endolipids are more similar than those that show inverse expression.

Polyunsaturated fatty acid (PUFA) metabolism was also broadly altered, with several long-chain PUFAs accumulating in IMQ-treated skin while decreasing in plasma. Altered PUFA metabolism has similarly been reported in human psoriasis, including changes in circulating n-3 and n-6 PUFAs and enrichment of PUFA-derived lipid mediators in lesional skin (Sorokin et al., 2018a; Simard et al., 2022). While n-3 PUFAs such as EPA and DHA are generally associated with anti-inflammatory and pro-resolving signaling, their elevation in inflamed skin may reflect a compensatory response rather than a direct pro-inflammatory effect; however, the functional significance of their accumulation in this model remains to be determined. Two notable endolipids that did not change in either skin or plasma following chronic IMQ treatment were corticosterone and PGE_2_. Although PGE_2_ is known to regulate keratinocyte proliferation and differentiation and can enhance IL-23/IL-17 signaling (Lee et al., 2019; Sheibanie et al., 2004), we did not observe significant alterations in its levels in either compartment. This is somewhat unexpected given the associated increase in arachidonic acid (AA) and elevated expression of COX-2 in the skin. However, this pattern is consistent with human psoriasis lipidomics, where HETE species, particularly 12-HETE, are more prominently altered than prostaglandins (Sorokin et al., 2018a; Sorokin et al., 2018b). In line with this, we observed increased 12-HETE levels, which is biologically plausible given its role as a major epidermal AA metabolite with chemotactic and pro-inflammatory functions in the skin (Kendall et al., 2015). These lipidomic findings suggest that IMQ-induced inflammation does not simply alter endolipid abundance but may also reshape the enzymatic and receptor systems that regulate endolipid signaling. We therefore examined whether these metabolite changes were accompanied by coordinated transcriptional alterations in whole skin and across individual skin compartments.

### 4.3 Transcriptional outcomes from the IMQ model elucidate novel enzyme and receptor regulatory processes

The whole-skin RT-qPCR data revealed a clear transcriptional pattern: expression of the major biosynthetic enzymes NAPE-PLD, DAGLα, and DAGLβ decreased, FAAH and MAGL remained unchanged, and CNR1, but not CNR2, was reduced. This pattern was directionally consistent with the public RNA-seq dataset from IMQ-treated mice (Swindell et al., 2017), supporting its reproducibility. The discrepancy between reduced biosynthetic enzyme expression and increased tissue EC levels indicates that transcriptional changes alone do not predict tissue endolipid levels. NAPE-PLD and DAGLs represent major, but not exclusive, biosynthetic routes for NAEs and 2-AG, respectively, and alternative enzymatic pathways may contribute to their production (Ueda et al., 2013; Tsuboi et al., 2018; Murataeva et al., 2014). Indeed, NAPE-PLD-deficient cells can maintain NAE production through compensatory pathways, including under inflammatory conditions (Liu et al., 2006) and we showed previously that is a consistent, though significantly diminished level of NAEs throughout the CNS in the NAPE-PLD KO mice (Leishman et al., 2016). NAPE-PLD activity can also be regulated independently of its transcript abundance, including through direct modulation by bile acids (Margheritis et al., 2016), which is particularly relevant given the marked bile acid changes observed in our dataset. Similarly, FAAH and MAGL are key enzymes in the degradation of NAEs and 2-AG, respectively, while additional enzymes, including NAAA for selected NAEs and ABHD6/ABHD12 for 2-AG, also contribute to their metabolism (Ueda et al., 2013; Maccarrone et al., 2015; Blankman et al., 2007). Thus, endolipid concentrations reflect the combined activity of multiple biosynthetic and degradative pathways rather than expression of individual metabolic enzymes alone. Additional mechanisms, including altered enzyme activity or protein abundance, substrate availability, feedback regulation, and changes in cellular composition, may also contribute, although these possibilities remain speculative in the absence of direct protein or enzyme-activity measurements. Our epidermis-versus-dermis analyses from an independent cohort further revealed that these transcriptional changes are compartment-dependent. *Dagla* and *Daglb* downregulation was primarily driven by epidermal changes, whereas *Napepld* decreased in both layers. Notably, although *Faah* and *Mgll* were unchanged in whole skin, both transcripts were reduced in the separated epidermal and dermal compartments. Given the independent cohorts and different tissue-processing procedures, these datasets should be interpreted within their respective experimental frameworks. While CB2 has been reported to be increased in both human psoriatic lesions and at least one IMQ-based mouse study (Turk and Kirchhof, 2026; Li et al., 2022), our whole-skin analysis did not detect a net increase, consistent with the Swindell RNA-seq dataset (Swindell et al., 2017). This discrepancy may reflect differences in model conditions and, importantly, the fact that bulk skin measurements average across dynamically changing cell populations. In this context, our finding of decreased epidermal *Cnr2* but increased dermal *Cnr2* suggests that spatial redistribution of CB2 receptors, potentially reflecting reduced keratinocyte Cnr2 alongside increased immune cell number/expression, may be more informative and biologically meaningful in psoriasis.

To determine whether this remodeling extended beyond canonical ECS components, we next examined a broader panel of endolipid-responsive receptors, nuclear lipid sensors, auxiliary metabolic enzymes, and genes involved in intracellular lipid handling, bile acid metabolism, and inflammatory lipid signaling.

Beyond CB1 and CB2, our expanded panel suggests that psoriasiform inflammation also remodels non-classical ECS signaling pathways. TRPV4 expression was significantly reduced, whereas TRPV1 remained unchanged. TRP channels are increasingly recognized as important regulators of cutaneous barrier function, inflammation, and sensory signaling, and their modulation has been proposed as a potential therapeutic strategy in psoriasis (da Silva et al., 2026). This is particularly relevant to the ECS, as several cannabinoid, ECS-related, and many of the endolipids measured here can engage TRPV channels (Zygmunt et al., 1999; Bisogno et al., 2001; Huang et al., 2002; Di Meo et al., 2022; De Petrocellis et al., 2012; Raboune et al., 2014; Sokabe et al., 2022). The role of TRPV4 in psoriasis appears complex: reduced expression has been reported in human lesional skin (Turk and Kirchhof, 2026), whereas increased TRPV4 protein expression has been observed in human psoriatic keratinocytes, and genetic deletion or pharmacological inhibition of TRPV4 attenuated IMQ-induced skin inflammation (Amalia et al., 2023). TRPV4 has also been functionally implicated in psoriatic itch and neurocutaneous signaling (Zhang et al., 2025; De Logu et al., 2023). Together, these findings suggest that TRPV4 is functionally relevant to psoriasis, although its regulation and contribution likely depend on cellular and disease context (Moore, 2022). In parallel, *Gpr55* was markedly upregulated in our dataset. Although the role of GPR55 in psoriasis remains less well-defined than that of canonical cannabinoid receptors, GPR55 is activated by several cannabinoid-related lipids (Lauckner et al., 2008; De Petrocellis and Di Marzo, 2010) and has been linked to inflammatory signaling, including NF-κB-related pathways (Trigo et al., 2024); moreover, increased GPR55 expression has been reported in granulocytes from patients with psoriasis (Ambrożewicz et al., 2018).

The changes in nuclear lipid sensors were similarly coherent with a dysregulated anti-inflammatory/metabolic program. Both *Ppara* and *Pparg* expression were reduced, consistent with the reported downregulation of these receptors in human psoriatic lesions (Turk and Kirchhof, 2026) and with their established roles in regulating keratinocyte differentiation, lipid metabolism, and inflammatory processes (Schmuth et al., 2008; Blunder et al., 2021). This is particularly relevant because several endolipids elevated in our study, including AEA, PEA, OEA, and related species, can signal through PPAR pathways (O'Sulliv and an, 2007; Baswan et al., 2020), and activation of PPARγ has long been associated with anti-proliferative effects in keratinocytes and has therapeutic potential in psoriasis (Wilkinson and Williamson, 2007; Sobolev et al., 2020; Ellis et al., 2000; Morin et al., 2021). We also observed reduced *Nr1h4* (FXR) expression, which fits with emerging evidence that bile-acid/FXR signaling is protective in psoriasis-like inflammation: experimental enhancement of bile-acid signaling alleviates psoriasiform dermatitis and reduces IL-17-related inflammation (Shi et al., 2022; Chen et al., 2023). In contrast, ABHD6 and ABHD12, alternative 2-AG hydrolases, were not significantly altered, indicating they are unlikely to be major contributors in this model.

Finally, several changes pointed to broader remodeling of intracellular lipid handling, bile acid metabolism, and inflammatory lipid signaling. The increase in *Fabp5* is particularly relevant given growing evidence implicating FABP5 in psoriasis pathogenesis. FABP5 is elevated in patients with psoriasis and correlates with disease severity (Kozłowska et al., 2021), while recent studies suggest that it can promote psoriatic inflammation through keratinocyte ferroptosis (Mieczkowski et al., 2026). Moreover, the selective FABP5 inhibitor ART26.12 reduced inflammatory responses in reconstructed human epidermis and attenuated IMQ-induced psoriasiform dermatitis (Warren et al., 2025), supporting FABP5 as a functionally relevant and potentially druggable pathway in psoriasis. *Ppap2a*, which encodes a phosphatase involved in degrading lysophosphatidic acid (LPA), was reduced; although we did not measure LPA directly, this finding is interesting in light of recent work implicating LPA/LPAR5 signaling in IMQ-induced psoriasis-like inflammation (Kim et al., 2021; Lei et al., 2021), raising the possibility that reduced LPA degradation may contribute to inflammatory signaling. The bile acid-related genes were particularly notable: *Cyp8b1* and *Abcb11* were markedly upregulated, whereas *Cyp27a1* was downregulated, indicating reprogramming of bile-acid synthesis and transport. Given that *Abcb11* encodes the bile salt export pump (BSEP) and that our lipidomics showed decreased cholic acid but increased deoxycholic and taurocholic acid species, these transcriptional changes are consistent with a shift in bile-acid handling rather than a passive bystander effect.

### 4.4 Limitations, conclusions, and future directions

Several limitations of this study should be acknowledged. First, our analyses were performed at a single time point (day 7), reflecting sustained inflammation but not capturing early dynamic changes during disease initiation and progression. Second, although we complemented whole-skin measurements with epidermis/dermis separation, these compartment-specific analyses were conducted in an independent cohort with a smaller sample size, involved different tissue-processing procedures, and were not directly paired with lipidomic measurements, limiting direct integration across datasets. Third, transcriptional measurements do not necessarily reflect enzymatic activity or lipid flux, and functional studies will be required to determine how these pathways contribute to endolipid regulation in inflamed skin. Finally, while the IMQ model recapitulates many key features of human psoriasis, including IL-23/IL-17-driven inflammation and epidermal hyperproliferation, it remains an acute murine model and may not fully capture the complexity and chronicity of human disease. Future studies integrating comprehensive lipidomic and transcriptomic analyses of human psoriatic skin will be essential to determine the extent to which alterations observed in the IMQ model recapitulate the human endocannabinoid landscape and to improve the translational evaluation of endocannabinoid-targeted therapeutic strategies.

Overall, our findings support the concept that the endocannabinoid system and related endolipids represent an important regulatory network in psoriatic skin inflammation. Elevated endolipid levels in psoriatic skin, together with transcriptional alterations in ECS-related proteins, lipid transporters, and bile acid pathways, point toward a coordinated metabolic adaptation within inflamed tissue. These observations are particularly relevant in light of the growing interest in therapies targeting endolipid signaling, including synthetic and phytocannabinoids, CB2-selective agonists (He et al., 2023), enzyme inhibitors targeting FAAH or MAGL (Lin et al., 2026), FABP5 inhibitors (Warren et al., 2025), and multifunctional compounds such as CB2/PPARγ agonists (Río et al., 2018; Scheau et al., 2020). By integrating lipidomic and transcriptional datasets across skin and plasma, this study provides a comprehensive molecular resource for the widely used IMQ-induced psoriasiform dermatitis model and identifies multiple endolipid-responsive pathways that may represent actionable targets for therapeutic intervention, warranting further validation in human disease.

## Data Availability

The datasets presented in this study can be found in online repositories. The names of the repository/repositories and accession number(s) can be found in the article/Supplementary Material.

## Supplementary material

SUPPLEMENTARY FIGURE S1 Lack of correlation between skin and plasma imiquimod (IMQ) concentrations. IMQ concentrations were measured in skin and plasma samples collected 24 h after the final topical application. Correlation analysis revealed no significant relationship between skin and plasma IMQ levels across individual animals (n = 9), as assessed by both Pearson (r = −0.13, p = 0.74) and Spearman (r = 0.12, p = 0.76) correlation tests. These data indicate that systemic exposure to IMQ does not directly reflect local skin concentrations under these experimental conditions.

SUPPLEMENTARY FIGURE S2 Data analysis of all endolipids scanned in skin and plasma. The tables list the analytical data of the IMQ versus control comparisons. See Methods for description of Python data analysis for fold change (denoted by arrows) and t-tests (colors denote significance levels and direction) to generate the heatmap colors and arrows. Dark green represents significant increases after treatment (p ≤ 0.05), while light green represents significance levels of p ≤ 0.1–0.05. Dark orange represents significant decreases after treatment (p ≤ 0.05), while light orange represents significance levels of p ≤ 0.1–0.05. Fold change is indicated by the number of arrows, where 1 arrow corresponds to a 1–1.49-fold difference, 2 arrows to a 1.5–1.99-fold difference, 3 arrows to a 2–2.99-fold difference, 4 arrows to a 3–9.99-fold difference, and 5 arrows to a difference of tenfold or more. BAL (below analytical limits) refers to endolipids that were measured in at least one sample, but less than 4, making statistical analysis unreliable. BDL (below detectable limits) refers to endolipids that were not detected in any samples. Blank cells indicate that endolipids were present in at least 4 samples in each treatment group and that no significant differences were present. Abbreviations: GABA, γ-aminobutyric acid; HETE, hydroxyeicosatetraenoic acid; IMQ, imiquimod; TUDCA, tauroursodeoxycholic acid; TDCA, taurodeoxycholic acid; TCDCA, taurochenodeoxycholic acid; GUDCA, glycoursodeoxycholic acid; GDCA, glycodeoxycholic acid; GCDCA, glycochenodeoxycholic acid; TLCA, taurolithocholic acid; GLCA, glycolithocholic acid.
