# Supplementary material for: Integrated lipidomic and transcriptomic profiling of imiquimod-induced psoriasiform dermatitis
Source: Front Cell Dev Biol. 2026 Sep 25;14:1881695. doi: 10.3389/fcell.2026.1881695 (PMC13649676; doi:10.3389/fcell.2026.1881695)
Supplement: Supplementary file 1 [file DataSheet1.PDF]

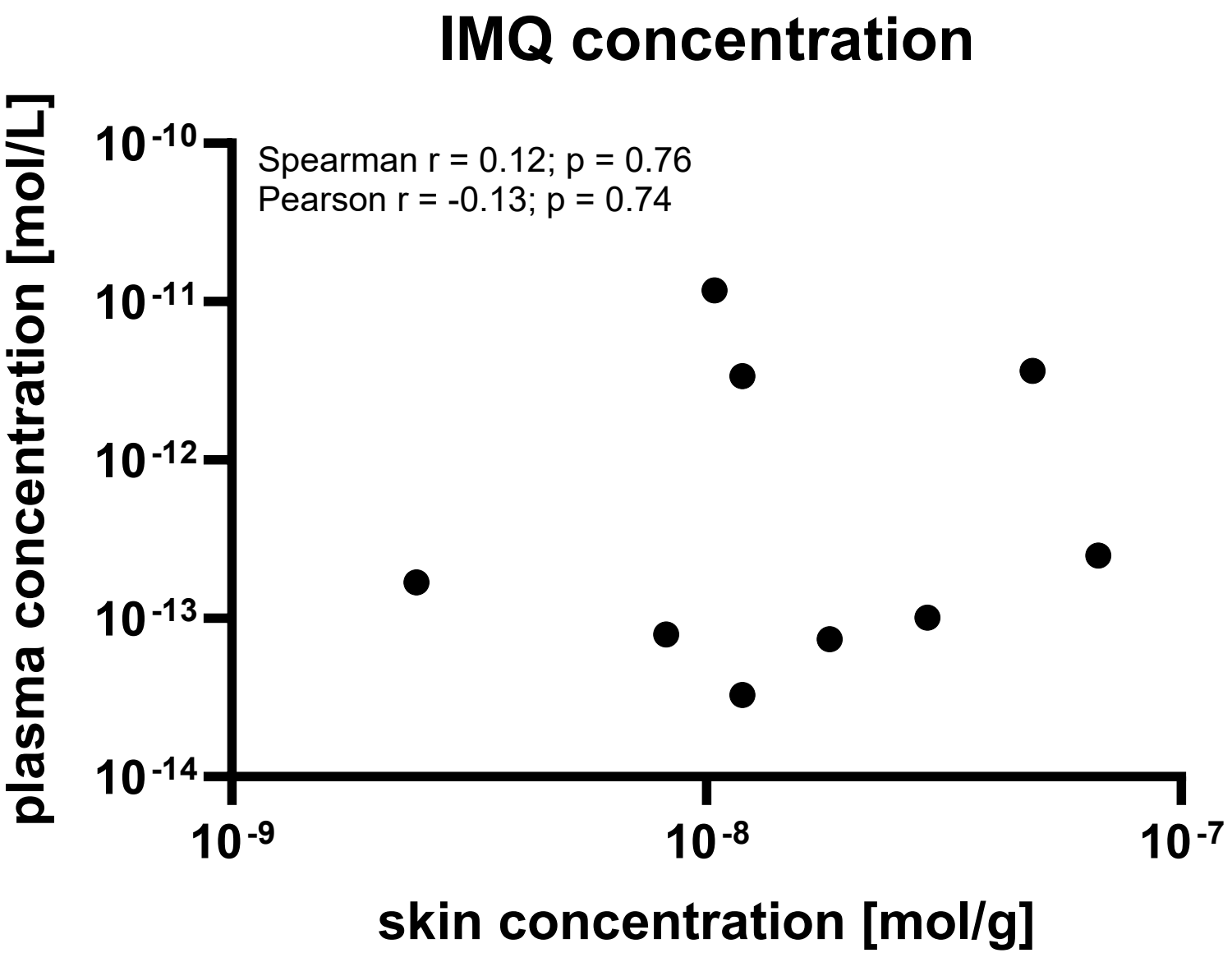
IMQ concentration
Spearman r = 0.12; p = 0.76
Pearson r = -0.13; p = 0.74
plasma concentration [mol/L]
10^-10
10^-11
10^-12
10^-13
10^-14
10^-9
10^-8
10^-7
skin concentration [mol/g]
