# Supplementary material for: Integrated lipidomic and transcriptomic profiling of imiquimod-induced psoriasiform dermatitis
Source: Front Cell Dev Biol. 2026 Sep 25;14:1881695. doi: 10.3389/fcell.2026.1881695 (PMC13649676; doi:10.3389/fcell.2026.1881695)
Supplement: Supplementary file 2 [file DataSheet2.PDF]

	SKIN				
	Mean in Control [mol/g]	Mean in IMQ [mol/g]	Heatmap	Fold Change	p-value
			IMQ compared to Control		
**Oleic acid**	3.80E-07	1.63E-05	↑↑↑↑↑	4.30E+01	7.32E-07
**Linoleic acid**	5.38E-07	9.31E-07	↑↑	1.73E+00	4.93E-04
**Arachidonic acid**	2.28E-07	5.64E-07	↑↑↑	2.48E+00	7.69E-06
**Docosahexaenoic acid**	3.79E-08	8.28E-08	↑↑↑	2.18E+00	1.55E-05
**Stearidonic acid**			BDL	BDL	BDL
**Eicosapentaenoic acid**	2.51E-09	5.03E-09	↑↑↑	2.00E+00	6.93E-05
**2-oleoyl glycerol**	1.28E-08	3.73E-08	↑↑↑	2.92E+00	8.89E-08
**2-linoleoyl glycerol**	8.54E-09	2.31E-08	↑↑↑	2.70E+00	1.57E-08
**2-arachidonoyl glycerol**	7.80E-09	2.66E-08	↑↑↑↑	3.40E+00	1.93E-10
***N-* palmitoyl ethanolamine**	2.56E-09	5.96E-08	↑↑↑↑↑	2.33E+01	2.22E-07
***N* -stearoyl ethanolamine**	2.81E-09	5.64E-08	↑↑↑↑↑	2.01E+01	3.56E-09
***N* -oleoyl ethanolamine**	1.08E-09	6.11E-09	↑↑↑↑	5.66E+00	4.43E-06
***N* -linoleoyl ethanolamine**	1.65E-10	3.20E-09	↑↑↑↑↑	1.94E+01	1.36E-06
***N-* arachidonoyl ethanolamine**	3.26E-11	3.41E-10	↑↑↑↑↑	1.05E+01	2.03E-05
***N-* docosahexaenoyl ethanolamine**	1.10E-11	1.96E-11	↑↑	1.78E+00	7.34E-06
**N-eicosapentaneoyl ethanolamine**			BDL	BDL	BDL
***N-* palmitoyl alanine**	9.43E-11	1.76E-10	↑↑	1.86E+00	6.89E-02
***N-* stearoyl alanine**	2.44E-11	2.73E-10	↑↑↑↑↑	1.12E+01	3.19E-04
***N-* oleoyl alanine**	4.02E-12	2.29E-11	↑↑↑↑	5.70E+00	1.88E-05
***N-* linoleoyl alanine**	7.17E-12	7.36E-12		1.03E+00	8.77E-01
***N-* arachidonoyl alanine**		4.08E-12	BAL	BAL	BAL
***N-* docosahexaenoyl alanine**	2.12E-12	2.13E-12		1.01E+00	9.80E-01
***N-* palmitoyl GABA**	3.43E-12	7.21E-12		2.10E+00	1.15E-01
***N-* stearoyl GABA**	1.17E-11	1.24E-11		1.06E+00	7.67E-01
***N-* oleoyl GABA**	3.70E-12	4.42E-12		1.20E+00	4.80E-01
***N-* linoleoyl GABA**			BDL	BDL	BDL
***N-* arachidonoyl GABA**			BDL	BDL	BDL
***N-* docosahexaenoyl GABA**			BDL	BDL	BDL
***N-* palmitoyl glycine**	1.87E-10	2.20E-10		1.18E+00	3.21E-01
***N-* stearoyl glycine**	1.24E-10	4.75E-10	↑↑↑↑	3.84E+00	3.65E-05
***N-* oleoyl glycine**	2.70E-11	7.09E-11	↑↑↑	2.63E+00	2.81E-04
***N-* linoleoyl glycine**	1.99E-11	2.71E-11		1.36E+00	1.79E-01
***N-* arachidonoyl glycine**	5.19E-12	7.72E-12	↑	1.49E+00	2.65E-02
***N-* docosahexaenoyl glycine**	2.47E-11	1.79E-11		-1.38E+00	1.73E-01

	SKIN				
	Mean in Control [mol/g]	Mean in IMQ [mol/g]	Heatmap	Fold Change	p-value
			IMQ compared to Control		
***N*-palmitoyl leucine**	1.33E-11	5.45E-11	↑↑↑↑	4.11E+00	1.54E-05
***N*-stearoyl leucine**	8.35E-12	1.29E-10	↑↑↑↑↑	1.54E+01	9.08E-08
***N*-oleoyl leucine**	6.20E-12	1.68E-11	↑↑↑	2.71E+00	2.13E-03
***N*-linoleoyl leucine**	1.75E-11	1.25E-11	↓	-1.40E+00	3.11E-02
***N*-arachidonoyl leucine**	3.10E-12	2.99E-12	BAL	BAL	BAL
***N*-docosahexaenoyl leucine**	1.16E-11	3.76E-11	↑↑↑↑	3.24E+00	4.30E-09
***N*-palmitoyl methionine**	2.83E-11	3.22E-11		1.14E+00	3.02E-01
***N*-stearoyl methionine**		3.31E-11	BAL	BAL	BAL
***N*-oleoyl methionine**	5.69E-12	6.82E-12	BAL	BAL	BAL
***N*-linoleoyl methionine**	3.83E-12	3.65E-12	BAL	BAL	BAL
***N*-arachidonoyl methionine**			BDL	BDL	BDL
***N*-docosahexaenoyl methionine**			BDL	BDL	BDL
***N*-palmitoyl phenylalanine**	1.65E-11	5.55E-11	↑↑↑↑	3.38E+00	1.35E-05
***N*-stearoyl phenylalanine**	6.60E-12	1.01E-10	↑↑↑↑↑	1.52E+01	9.58E-08
***N*-oleoyl phenylalanine**	5.45E-12	1.41E-11	↑↑↑	2.59E+00	1.57E-03
***N*-linoleoyl phenylalanine**	2.85E-12	3.78E-12		1.33E+00	1.93E-01
***N*-arachidonoyl phenylalanine**	5.60E-12	4.95E-12		-1.13E+00	5.91E-01
***N*-docosahexaenoyl phenylalanine**	3.90E-12	5.93E-12	↑↑	1.52E+00	5.94E-03
***N*-palmitoyl proline**	2.13E-10	9.84E-11	↓↓↓	-2.16E+00	1.16E-04
***N*-stearoyl proline**	1.19E-10	1.92E-10	↑↑	1.62E+00	6.02E-03
***N*-oleoyl proline**	2.60E-11	2.51E-11		-1.03E+00	7.79E-01
***N*-linoleoyl proline**			BDL	BDL	BDL
***N*-arachidonoyl proline**			BDL	BDL	BDL
***N*-docosahexaenoyl proline**			BDL	BDL	BDL
***N*-palmitoyl serine**	5.24E-11	1.37E-10	↑↑↑	2.62E+00	2.17E-06
***N*-stearoyl serine**	5.15E-11	4.60E-10	↑↑↑↑	8.93E+00	3.82E-07
***N*-oleoyl serine**	1.88E-11	3.55E-11	↑↑	1.89E+00	1.75E-04
***N*-linoleoyl serine**	5.59E-12	1.31E-11	↑↑↑	2.35E+00	1.49E-03
***N*-arachidonoyl serine**			BDL	BDL	BDL
***N*-docosahexaenoyl serine**			BDL	BDL	BDL
***N*-palmitoyl taurine**	8.15E-10	2.75E-09	↑↑↑↑	3.37E+00	9.17E-11
***N*-stearoyl taurine**	2.77E-10	4.34E-09	↑↑↑↑↑	1.57E+01	7.56E-09
***N*-oleoyl taurine**	3.81E-10	1.93E-09	↑↑↑↑	5.06E+00	4.34E-09
***N*-arachidonoyl taurine**	1.39E-10	1.82E-10	BAL	BAL	BAL

	SKIN				
	Mean in Control [mol/g]	Mean in IMQ [mol/g]	Heatmap	Fold Change	p-value
			IMQ compared to Control		
N- **palmitoyl tryptophan**	1.27E-11	2.12E-11	↑↑	1.66E+00	1.92E-02
N- **stearoyl tryptophan**			BDL	BDL	BDL
N- **oleoyl tryptophan**	8.52E-12	1.63E-11	↑↑	1.91E+00	8.16E-02
N- **linoleoyl tryptophan**			BDL	BDL	BDL
N- **arachidonoyl tryptophan**			BDL	BDL	BDL
N- **docosahexaenoyl tryptophan**			BDL	BDL	BDL
N- **palmitoyl tyrosine**	3.20E-12	1.48E-11	↑↑↑↑	4.62E+00	1.42E-02
N- **stearoyl tyrosine**	5.58E-12	6.20E-11	BAL	BAL	BAL
N- **oleoyl tyrosine**		1.95E-11	BAL	BAL	BAL
N- **linoleoyl tyrosine**		2.96E-11	BAL	BAL	BAL
N- **arachidonoyl tyrosine**			BDL	BDL	BDL
N- **docosahexaenoyl tyrosine**		1.16E-11	BAL	BAL	BAL
N- **palmitoyl valine**	6.36E-11	3.71E-10	↑↑↑↑	5.84E+00	3.89E-04
N- **stearoyl valine**	3.77E-11	7.06E-10	↑↑↑↑↑	1.87E+01	2.15E-05
N- **oleoyl valine**	6.40E-11	1.34E-10	↑↑↑	2.09E+00	1.19E-03
N- **linoleoyl valine**	1.32E-11	8.21E-12	↓↓	-1.61E+00	1.37E-02
N- **arachidonoyl valine**	1.74E-12	2.26E-12		1.30E+00	1.17E-01
N- **docosahexaenoyl valine**	2.86E-12	3.11E-12		1.09E+00	7.56E-01
**Prostaglandin E2**	3.91E-09	4.71E-09		1.20E+00	3.51E-01
**Prostaglandin F2a**	1.28E-09	1.71E-09	↑	1.34E+00	1.64E-04
**12(S)-HETE**	2.59E-10	2.53E-09	↑↑↑↑	9.76E+00	4.87E-04
**Cholic acid**	3.01E-10	1.01E-10	↓↓↓	-2.97E+00	4.32E-02
**Deoxycholic acid**	2.34E-10	3.72E-10	↑↑	1.59E+00	3.73E-02
**Glycocholic acid**	6.15E-12	5.99E-12		-1.03E+00	9.34E-01
**Taurocholic acid**	6.32E-10	3.27E-09	↑↑↑↑	5.18E+00	2.03E-05
**TUDCA**	2.56E-11	7.73E-11	BAL	BAL	BAL
**TDCA+TCDCA**	2.25E-11	7.79E-11	↑↑↑↑	3.47E+00	4.78E-03
**GUDCA**	1.89E-12	2.95E-12		1.56E+00	1.41E-01
**GDCA+GCDCA**	2.34E-11	1.31E-11		-1.78E+00	2.92E-01
**TLCA**	7.67E-12	8.83E-12	BAL	BAL	BAL
**GLCA**	1.37E-11		BAL	BAL	BAL
**Hydrocortisone**			BDL	BDL	BDL
**Corticosterone**			BDL	BDL	BDL
**IMQ**		3.58E-08	NA	NA	NA

	PLASMA				
	Mean in Control [mol/L]	Mean in IMQ [mol/L]	Heatmap	Fold Change	p-value
			IMQ compared to Control		
**Oleic acid**	6.49E-08	3.21E-08	↓↓↓	-2.02E+00	4.05E-09
**Linoleic acid**	9.65E-08	5.96E-08	↓↓	-1.62E+00	2.98E-09
**Arachidonic acid**	8.73E-09	6.46E-09	↓	-1.35E+00	1.67E-04
**Docosahexaenoic acid**	9.39E-09	5.68E-09	↓↓	-1.65E+00	1.27E-06
**Stearidonic acid**	2.20E-10	8.68E-11	↓↓↓	-2.53E+00	1.11E-08
**Eicosapentaenoic acid**	9.26E-10	4.83E-10	↓↓	-1.92E+00	1.26E-04
**2-oleoyl glycerol**	3.71E-09	2.65E-09	↓	-1.40E+00	1.46E-03
**2-linoleoyl glycerol**	8.05E-09	4.43E-09	↓↓	-1.82E+00	1.42E-08
**2-arachidonoyl glycerol**	1.41E-10	9.61E-11	↓	-1.47E+00	2.82E-05
***N*- palmitoyl ethanolamine**	7.66E-12	5.27E-12	↓	-1.45E+00	5.94E-07
***N*-stearoyl ethanolamine**	1.64E-12	1.37E-12	↓	-1.20E+00	1.68E-04
***N*-oleoyl ethanolamine**	4.67E-12	3.36E-12	↓	-1.39E+00	7.58E-06
***N*-linoleoyl ethanolamine**	5.94E-12	4.48E-12	↓	-1.32E+00	1.73E-03
***N*- arachidonoyl ethanolamine**	3.07E-12	2.01E-12	↓↓	-1.53E+00	1.94E-07
***N*- docosahexaenoyl ethanolamine**	1.07E-12	6.80E-13	↓↓	-1.58E+00	2.40E-08
**N-eicosapentaneoyl ethanolamine**	2.69E-14	2.56E-14		-1.05E+00	7.84E-01
***N*- palmitoyl alanine**	5.41E-13	3.44E-13	↓↓	-1.57E+00	7.62E-03
***N*- stearoyl alanine**	9.85E-14	3.07E-13	↑↑↑↑	3.12E+00	3.14E-02
***N*- oleoyl alanine**	4.34E-13	2.16E-13	↓↓↓	-2.01E+00	1.23E-05
***N*- linoleoyl alanine**	4.58E-13	2.49E-13	↓↓	-1.84E+00	6.87E-05
***N*- arachidonoyl alanine**	1.14E-13	6.09E-14	↓↓	-1.87E+00	9.94E-02
***N*- docosahexaenoyl alanine**	1.72E-13	8.29E-14	↓↓↓	-2.07E+00	5.48E-03
***N*- palmitoyl GABA**	7.88E-14	6.35E-14		-1.24E+00	3.76E-01
***N*- stearoyl GABA**	1.95E-13	1.68E-13	BAL	BAL	BAL
***N*- oleoyl GABA**			BDL	BDL	BDL
***N*- linoleoyl GABA**			BDL	BDL	BDL
***N*- arachidonoyl GABA**			BDL	BDL	BDL
***N*- docosahexaenoyl GABA**	6.49E-14	7.61E-14	BAL	BAL	BAL
***N*- palmitoyl glycine**	1.06E-12	5.93E-12	↑↑↑↑	5.58E+00	1.23E-05
***N*- stearoyl glycine**	6.51E-13	2.71E-11	↑↑↑↑↑	4.16E+01	1.48E-09
***N*- oleoyl glycine**	6.23E-13	7.70E-13	↑	1.24E+00	4.89E-02
***N*- linoleoyl glycine**	9.41E-13	6.71E-13	↓	-1.40E+00	6.38E-04
***N*- arachidonoyl glycine**	8.62E-14	7.32E-14		-1.18E+00	3.18E-01
***N*- docosahexaenoyl glycine**	7.22E-13	3.67E-13	↓↓	-1.97E+00	1.08E-03

	PLASMA				
	Mean in Control [mol/L]	Mean in IMQ [mol/L]	Heatmap	Fold Change	p-value
			IMQ compared to Control		
***N*- palmitoyl leucine**	2.69E-13	2.02E-13	↓	-1.33E+00	6.70E-02
***N*- stearoyl leucine**	2.45E-14	8.29E-12	↑↑↑↑↑	3.39E+02	2.30E-12
***N*- oleoyl leucine**	1.69E-13	1.46E-13		-1.16E+00	3.84E-01
***N*- linoleoyl leucine**	5.33E-13	4.66E-13		-1.14E+00	2.67E-01
***N*- arachidonoyl leucine**	5.17E-14	6.79E-14	↑	1.31E+00	5.83E-02
***N*- docosahexaenoyl leucine**	2.93E-13	3.23E-13		1.10E+00	2.32E-01
***N*- palmitoyl methionine**	2.41E-13	1.26E-13	↓↓	-1.91E+00	1.97E-02
***N*- stearoyl methionine**	1.85E-13	1.70E-12	↑↑↑↑	9.20E+00	7.94E-08
***N*- oleoyl methionine**	3.40E-13	2.22E-13	↓↓	-1.53E+00	5.80E-04
***N*- linoleoyl methionine**	5.29E-13	3.07E-13	↓↓	-1.72E+00	6.95E-04
***N*- arachidonoyl methionine**	7.64E-14	6.34E-14		-1.21E+00	3.73E-01
***N*- docosahexaenoyl methionine**	1.44E-13	9.43E-14	↓↓	-1.53E+00	7.24E-03
***N*- palmitoyl phenylalanine**	3.33E-13	2.37E-13	↓	-1.40E+00	1.06E-02
***N*- stearoyl phenylalanine**	1.06E-13	4.80E-12	↑↑↑↑↑	4.55E+01	2.23E-12
***N*- oleoyl phenylalanine**	1.83E-13	1.69E-13		-1.08E+00	5.70E-01
***N*- linoleoyl phenylalanine**	2.52E-13	1.74E-13	↓	-1.45E+00	1.42E-03
***N*- arachidonoyl phenylalanine**	1.12E-13	9.57E-14		-1.17E+00	1.37E-01
***N*- docosahexaenoyl phenylalanine**	1.62E-13	1.47E-13		-1.10E+00	4.21E-01
***N*- palmitoyl proline**	8.40E-13	1.01E-12		1.21E+00	3.07E-01
***N*- stearoyl proline**	8.41E-13	1.79E-12	↑↑↑	2.12E+00	2.46E-05
***N*- oleoyl proline**			BDL	BDL	BDL
***N*- linoleoyl proline**			BDL	BDL	BDL
***N*- arachidonoyl proline**			BDL	BDL	BDL
***N*- docosahexaenoyl proline**			BDL	BDL	BDL
***N*- palmitoyl serine**	2.14E-13	2.07E-13		-1.04E+00	7.99E-01
***N*- stearoyl serine**	2.03E-13	2.39E-13		1.18E+00	5.01E-01
***N*- oleoyl serine**	1.79E-13	9.90E-14	↓↓	-1.81E+00	5.27E-02
***N*- linoleoyl serine**			BDL	BDL	BDL
***N*- arachidonoyl serine**			BDL	BDL	BDL
***N*- docosahexaenoyl serine**			BDL	BDL	BDL
***N*- palmitoyl taurine**	1.49E-11	1.19E-10	↑↑↑↑	7.99E+00	6.30E-11
***N*- stearoyl taurine**	4.40E-12	2.42E-10	↑↑↑↑↑	5.49E+01	1.77E-10
***N*- oleoyl taurine**	1.43E-11	2.82E-11	↑↑	1.97E+00	1.49E-08
***N*- arachidonoyl taurine**	3.22E-12	3.14E-12		-1.03E+00	5.92E-01

	PLASMA				
	Mean in Control [mol/L]	Mean in IMQ [mol/L]	Heatmap	Fold Change	p-value
			IMQ compared to Control		
***N*- palmitoyl tryptophan**	2.95E-13	3.75E-13		1.27E+00	1.91E-01
***N*- stearoyl tryptophan**	1.89E-13	2.08E-13		1.10E+00	4.21E-01
***N*- oleoyl tryptophan**	7.05E-13	4.10E-13	↓↓	-1.72E+00	1.06E-03
***N*- linoleoyl tryptophan**	4.73E-13	6.81E-13	↑	1.44E+00	4.00E-02
***N*- arachidonoyl tryptophan**			BDL	BDL	BDL
***N*- docosahexaenoyl tryptophan**			BDL	BDL	BDL
***N*- palmitoyl tyrosine**	3.87E-13	1.92E-13	↓↓↓	-2.01E+00	6.93E-03
***N*- stearoyl tyrosine**	2.02E-13	6.32E-13	↑↑↑↑	3.13E+00	1.49E-03
***N*- oleoyl tyrosine**	3.69E-13	2.29E-13	↓↓	-1.61E+00	2.76E-03
***N*- linoleoyl tyrosine**	5.09E-13	1.73E-13	↓↓↓	-2.94E+00	2.39E-04
***N*- arachidonoyl tyrosine**	5.71E-14	6.27E-14	BAL	BAL	BAL
***N*- docosahexaenoyl tyrosine**	3.85E-13	2.67E-13	↓	-1.44E+00	8.97E-03
***N*- palmitoyl valine**	1.03E-12	6.34E-13	↓↓	-1.62E+00	6.06E-03
***N*- stearoyl valine**	2.09E-13	5.12E-12	↑↑↑↑↑	2.45E+01	2.21E-15
***N*- oleoyl valine**	1.18E-12	1.03E-12		-1.14E+00	2.86E-01
***N*- linoleoyl valine**	3.20E-13	2.36E-13	↓	-1.36E+00	2.75E-03
***N*- arachidonoyl valine**	1.97E-14	3.01E-14		1.53E+00	1.28E-01
***N*- docosahexaenoyl valine**	1.49E-13	1.02E-13	↓	-1.46E+00	2.93E-02
**Prostaglandin E2**	1.98E-12	1.82E-12		-1.09E+00	5.15E-01
**Prostaglandin F2a**	9.82E-12	8.77E-12		-1.12E+00	1.81E-01
**12(S)-HETE**	9.45E-10	4.38E-10	↓↓↓	-2.16E+00	6.09E-02
**Cholic acid**	9.31E-10	8.76E-11	↓↓↓↓↓	-1.06E+01	3.00E-03
**Deoxycholic acid**	1.41E-10	1.39E-10		-1.02E+00	9.47E-01
**Glycocholic acid**	4.29E-12	2.98E-12		-1.44E+00	4.83E-01
**Taurocholic acid**	6.35E-10	2.12E-09	↑↑↑↑	3.33E+00	1.89E-05
**TUDCA**	4.98E-11	8.63E-11	↑↑	1.73E+00	1.63E-02
**TDCA+TCDCA**	2.90E-11	9.12E-11	↑↑↑↑	3.15E+00	9.30E-06
**GUDCA**			BDL	BDL	BDL
**GDCA+GCDCA**			BDL	BDL	BDL
**TLCA**	7.49E-13	6.10E-13		-1.23E+00	5.60E-01
**GLCA**			BDL	BDL	BDL
**Hydrocortisone**			BDL	BDL	BDL
**Corticosterone**	1.43E-10	1.66E-10		1.17E+00	3.30E-01
**IMQ**		2.05E-12	NA	NA	NA
